# Sensor Inputs and Closed-Loop Neuromodulation in Spinal Cord Injury: From Animal Models to Clinical Translation

**DOI:** 10.3390/s26144480

**Published:** 2026-07-15

**Authors:** Erika Rosado, Ahnsei Shon, Wei Wu

**Affiliations:** 1 Department of Anatomical Sciences and Neurobiology, University of Louisville School of Medicine, Louisville, KY 40202, USA; erika.rosado@louisville.edu; 2 Department of Neurological Surgery, University of Louisville School of Medicine, Louisville, KY 40202, USA; ahnsei.shon@louisville.edu; 3 Kentucky Spinal Cord Injury Research Center, University of Louisville, Louisville, KY 40202, USA

**Keywords:** spinal cord injury, closed-loop neuromodulation, epidural stimulation, transcutaneous spinal stimulation, functional electrical stimulation, electromyography, gait phase detection, wearable sensors

## Abstract

Closed-loop neuromodulation systems for spinal cord injury (SCI) comprise a sensor layer that detects motor state or intent, a controller that converts this information into stimulation commands, and a stimulation interface that modulates spinal, peripheral, or supraspinal circuits. Although neuromodulation has shown potential for improving stepping, standing, trunk control, and upper-limb function after SCI, the performance of closed-loop systems depends critically on their reliability, latency, and practicality. This structured narrative review focuses on the sensor inputs used in closed-loop neuromodulation for SCI, including kinematic sensors, electromyography, force and pressure sensors, vision-based sensing, and emerging neural interfaces. We summarize how these signals have been used to estimate gait phase, posture, motor intent, and task state, and how sensor-driven strategies have progressed from animal models to early clinical applications. Preclinical studies provide mechanistic insights into activity-dependent, phase-specific, and proprioceptive feedback-mediated control, whereas human studies suggest that sensor-guided stimulation may improve functional specificity; however, comparative evidence remains limited. Future translation will require robust multimodal sensing, standardized reporting of latency and calibration burden, and practical designs suitable for supervised clinical and, ultimately, home use.

## 1. Introduction

Paralysis after spinal cord injury (SCI) persists because lesions interrupt descending and ascending commands while leaving sublesional networks and sensory feedback circuits only partially accessible. Over the past decade, epidural and transcutaneous stimulation studies have shown that lumbosacral circuitry can still support stepping, standing, and task-specific motor output [1,2]. However, many early implementations were essentially open-loop: stimulation parameters were selected offline and then delivered continuously or according to simple therapist-defined rules. Although this approach can facilitate movement, it cannot adapt to rapid changes in gait phase, posture, loading, or voluntary effort [3,4,5]. In parallel, animal studies established why adaptive control matters. Targeted activity-dependent stimulation improves recovery more effectively than tonic stimulation, and spatiotemporal engagement of sensorimotor circuits can refine leg kinematics after SCI. These studies shifted the field from the idea of simply exciting the spinal cord toward the more effective concept of shaping spinal network activity in relation to ongoing behavior [4,6].

Clinical translation has accelerated with targeted epidural stimulation to restore overground walking, activity-specific programs that enable multiple motor behaviors, and, more recently, a brain–spine interface that supports natural walking in community settings. Upper-limb research has similarly progressed from mechanistic studies of cervical epidural stimulation to transcutaneous stimulation and multicenter evidence supporting arm and hand recovery [5,7,8,9,10,11]. The central question is not only whether neuromodulation works, but how sensing can make stimulation adaptive. An effective closed-loop system must detect motor state, extract a control variable, and update stimulation parameters with low latency and adequate reliability. Recent reviews emphasize the growing convergence of wearable sensing, AI, robotics, and neuromodulation; however, a concise SCI-focused synthesis centered on sensor choice and translational design remains warranted [12].

Here, we focus on studies in which the sensor or control signal changed the stimulation strategy, rather than cataloging all SCI neuromodulation studies.

## 2. Methods

This manuscript is a structured narrative review, not a systematic review or meta-analysis. The literature was identified through PubMed, Web of Science, and Google Scholar for English-language publications from January 2009 to March 2026. Search terms combined “spinal cord injury” or “SCI” with “closed-loop”, “activity-dependent”, “event-triggered”, “volition-controlled”, “brain-spine interface”, “epidural stimulation”, “transcutaneous spinal stimulation”, “functional electrical stimulation”, “EMG”, “wearable sensor”, “gait phase”, “walking”, “balance”, “trunk”, “upper limb”, and “hand”. Reference lists of key papers were also screened.

Studies were prioritized when they involved an animal or human SCI model/population, included a motor rehabilitation or motor-control endpoint, and used a sensor, biological signal, or decoded carrier to change stimulation timing, program selection, or parameters. Studies without simulation, non-SCI studies, robotics-only studies, reviews/opinion articles, and abstracts without full methodological descriptions were excluded from the primary evidence. Representative studies were selected based on mechanistic relevance, clinical importance, and the availability of controller information. Evidence was classified descriptively as preclinical mechanistic, preclinical translational, human feasibility/case report, human case series, controlled clinical study, or contextual rehabilitation evidence.

Control classes were defined as follows: real closed-loop, when a measured signal automatically updated stimulation during the task; event-triggered, when an event triggered a predefined stimulation burst; activity-dependent, when stimulation was paired with biological or movement-related activity; task-specific open-loop, when a predefined program was selected for a task without online sensor updating; therapist-triggered/manual, when a human operator adjusted stimulation based on observation; and continuous stimulation or clinical rehabilitation without a control sensor, when stimulation was delivered tonically or with therapy without online sensory control. The last three categories were retained as contextual clinical evidence but were not interpreted as direct evidence of real-time closed-loop sensor-stimulation control.

## 3. Sensor Inputs and Closed-Loop Control Concepts

**Figure 1:**
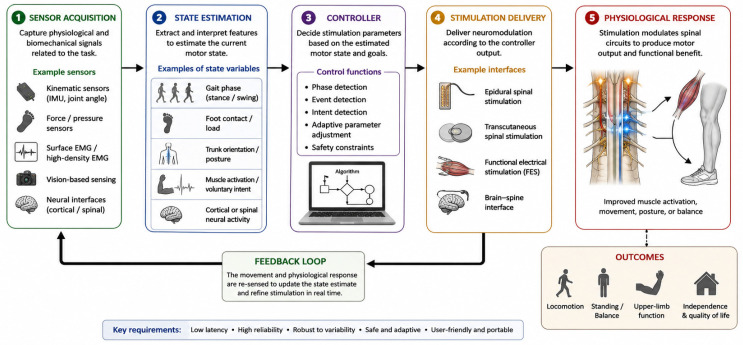
**Conceptual schematic of a closed-loop neuromodulation system for spinal cord injury.** Sensor inputs are acquired and processed in real time to estimate motor state, drive controller decisions, and deliver adaptive stimulation. Physiological and mechanical feedback is then re-sensed to update the state estimate and refine stimulation control.

A closed-loop neuromodulation system for SCI can be described in four steps: sensor acquisition, state estimation, controller decision, and stimulation delivery (Figure 1). The sensors do not need to capture every detail of movement. In most successful systems, they estimate a compact variable that is directly actionable, such as gait phase, foot contact, trunk tilt, or the onset of attempted movement. The operational categories used to classify studies are defined in Section 2 and applied in Table 1.

Although the sensing modalities are discussed separately below, they should not be interpreted as mutually exclusive approaches. In practical closed-loop neuromodulation systems, multimodal sensing is likely to be advantageous because different sensors provide complementary information. For example, kinematic sensors can estimate limb phase or trunk orientation, force and pressure sensors can confirm foot contact or loading, EMG can capture residual volitional effort or stimulation-evoked muscle responses, and vision-based systems can provide global movement context. Combining these inputs may improve state estimation, reduce false triggering, and make the controller more robust to dropout or degradation of any single sensor channel.

Multimodal sensing may be particularly important in SCI because the available control signal varies across individuals, tasks, and recovery stages. A patient with detectable residual EMG may benefit from intent-based gating, whereas another patient may require foot-contact, load, or trunk-orientation signals to guide stimulation timing. Similarly, treadmill stepping, overground walking, standing balance, and upper-limb control impose different sensing requirements. Hybrid sensor architectures may therefore support personalization by matching the sensing strategy to the functional goal and the preserved physiological signals.

At the same time, multimodal sensing introduces engineering challenges, including synchronization across sensors, increased calibration burden, higher processing load, and potential increases in end-to-end latency. A detailed review of sensor-fusion algorithms is beyond the scope of this article. Therefore, the present review discusses each sensing modality individually to clarify its specific strengths, limitations, and translational readiness, while recognizing that future clinically useful systems will likely combine multiple sensing inputs within task-specific control frameworks.

Most SCI systems rely on one of five sensor classes: kinematic sensors, force or pressure sensors, EMG, vision-based sensing, and neural recording. Kinematic signals from IMUs, goniometers, encoders, or motion-tracking systems are attractive because of their ability to directly capture gait phase, limb trajectory, and trunk orientation. Force and pressure sensors complement kinematics by identifying foot contact, stance loading, and weight shift, which are particularly useful for standing balance and gait transitions [5,7,21,22,23]. EMG is often the most physiologically informative signal because it reports both attempted volitional drive and evoked muscle responses. In tetraplegia, wearable sleeve arrays and high-density surface EMG can detect residual neural drive in muscles that appear clinically silent, while direct spinal cord–computer interfaces can convert those signals into control of a virtual or paralyzed hand [31,32,33]. For lower-limb rehabilitation, the control channel does not need to arise from the impaired limb itself. Tazoe et al. showed that preserved hand-muscle activity can be used to trigger and modulate stepping in people with paraplegia, enabling control of start–stop behavior, step length, and cadence through a non-invasive closed-loop interface [20].

Vision-based sensing is becoming increasingly relevant with camera systems that can extract joint kinematics without wearable hardware and can be paired with embedded classifiers. This approach is attractive for animal studies, laboratory gait analysis, and potentially home monitoring. Our recent *Sensors* study demonstrated an edge-AI framework that combined video-based joint tracking with on-device gait-phase classification to trigger phase-dependent neuromodulation in SCI mice [16]. The stimulation output can be delivered through epidural stimulation, transcutaneous spinal stimulation, or peripheral/functional electrical stimulation, depending on the task and the level of invasiveness that can be justified clinically. In practice, for gait-phase triggering, for example, a reliable foot-contact or trunk-angle signal may be more clinically useful than a complex classifier that fails during transitions [3,5,10,11,17]. The major sensor classes used in closed-loop SCI neuromodulation, including their typical variables, control roles, strengths, limitations, and representative studies, are summarized in Table 2.

## 4. Gait and Locomotion Recovery: From Animal Models to Human Translation

The mechanistic basis for closed-loop gait control was built largely in animal models. McPherson et al. showed that targeted activity-dependent spinal stimulation produced durable motor gains after chronic cervical SCI, supporting the principle that coupling stimulation to endogenous activity can shape plasticity. Wenger et al. extended this concept by delivering spatiotemporal stimulation patterns that engaged muscle synergies during locomotion, demonstrating that both the timing and spatial organization of stimulation can refine leg kinematics after SCI [4].

Nonhuman primate and rodent studies further clarified the role of sensor-driven control. Capogrosso et al. used cortical signals to trigger spinal stimulation in primates and alleviated gait deficits after SCI, providing a clear proof of concept for brain–spine control [13]. Moraud et al. showed that closed-loop regulation of trunk posture improves stepping by rebalancing proprioceptive feedback between the legs, and Bonizzato et al. later reported that a proportional brain-controlled spinal interface improved both immediate locomotor performance and long-term recovery in rats [14,15]. These preclinical studies collectively show that lower-limb neuromodulation may benefit from synchronization with behavior rather than tonic stimulation. They also show that state estimation does not always require a rich sensor suite; in some cases, a well-chosen single variable, such as cortical activity or trunk orientation, is sufficient to improve control.

Clinical gait studies provide a translational context for these preclinical concepts [1,2]. Gill et al. showed that lumbosacral neuromodulation can enable independent stepping after complete paraplegia, and Angeli et al. reported overground walking in selected individuals with chronic motor-complete SCI after epidural stimulation combined with intensive training [17,18]. Wagner et al. then demonstrated targeted spatiotemporal stimulation that supported adaptive overground walking under controlled conditions within days, shifting the field from generalized excitation of the cord to task-specific recruitment [5]. Subsequent studies increased both capability and ecological validity. Rowald et al. used activity-specific programs to rapidly restore standing, walking, cycling, swimming, and trunk control in people with complete paralysis [7]. Lorach et al. linked cortical signals to spinal stimulation through a brain–spine interface and enabled natural walking in a selected participant, providing perhaps the clearest example to date of an integrated sensor-controller-stimulator architecture in SCI rehabilitation [8].

Non-invasive intent-driven systems have also been developed. Selfslagh et al. explored a brain-controlled FES approach for locomotor rehabilitation, highlighting the broader potential of non-invasive intent decoding for gait assistance [19]. At the transcutaneous end of the field, stimulation combined with walking-based therapy is clinically feasible in motor-incomplete SCI, randomized feasibility data support pairing transcutaneous stimulation with locomotor training in subacute SCI, and multisite stimulation can improve walking and autonomic measures in motor-incomplete tetraplegia [21,22,23,34]. Most recently, Tazoe et al. demonstrated a non-invasive closed-loop spinal interface in which hand-muscle activity controlled start–stop stepping, step length, and cadence in people with paraplegia [20].

The gait literature shows that sensing adds value most clearly when stimulation must be matched to the phase or intent of movement. In the studies by Wenger and Wagner, stimulation was effective because its spatial and temporal patterns were linked to the locomotor cycle rather than delivered as tonic excitation. The clinical work of Gill, Angeli, Rowald, and Lorach extends this principle to human SCI: stimulation can enable stepping or walking, but performance improves when the stimulation program is aligned with the task being attempted. Preserved sensory feedback and residual descending drive remain important constraints, as shown by Formento and others [35]. For rhythmic treadmill stepping, a relatively simple gait-phase signal may be sufficient. For overground walking, start–stop transitions, variable terrain, or community use, however, the controller will likely need more than one input signal and a defined fallback strategy when the inferred gait state is uncertain. Representative animal and clinical studies are summarized in Table 1.

## 5. Standing, Trunk Control, and Balance

Standing and seated balance are attractive targets for closed-loop neuromodulation as the control objective is lower-dimensional than overground walking. Instead of coordinating many joints across the gait cycle, the system can focus on trunk angle, pelvic orientation, or center-of-pressure shifts. This translational logic is consistent with preclinical evidence that trunk posture is in itself a powerful control variable for locomotor performance [13]. Murphy et al. implemented a sensor-based threshold controller for right seated posture after SCI, demonstrating that real-time feedback to hip and trunk extensors can stabilize sitting [24]. Audu et al. subsequently explored posture-dependent control of stimulation in standing and later reported a neuroprosthesis for seated balance that used trunk-tilt information to drive functional neuromuscular stimulation [25,26]. Earlier implanted trunk-stimulation work by Triolo et al. had already demonstrated that restoring torso stability can improve reaching, respiration, and functional mobility in cervical SCI [27]. Non-invasive spinal stimulation has expanded these concepts. Rath et al. showed that transcutaneous spinal stimulation can improve trunk stability during sitting tasks after SCI, while Sayenko et al. reported that self-assisted standing was enabled by non-invasive spinal stimulation [28,29].

From a sensor perspective, balance applications may be especially promising because trunk angle, load symmetry, and pressure distribution can be measured reliably with relatively simple hardware. The challenge is safety: incorrect classification or delayed stimulation during standing carries a higher immediate fall risk than a mistimed pulse during supported stepping. That tradeoff argues for redundant sensing, conservative thresholds, and clear fail-safe behavior.

## 6. Upper-Limb Recovery

Upper-limb restoration presents a different control problem from gait. Arm and hand movements require finer spatial selectivity, more flexible timing, and stronger dependence on voluntary intent. Preclinical cervical epidural studies by Greiner et al. showed that lateral cervical electrodes can recruit upper-limb motoneurons segmentally through sensory afferents, establishing a mechanistic basis for selective control [9]. Barra et al. subsequently demonstrated that movement-phase-specific stimulation of cervical dorsal roots restores voluntary reach-and-grasp control in paralyzed monkeys, but only when residual descending signals are available to interact with the stimulation [30].

Clinical translation has progressed rapidly. Inanici et al. reported that transcutaneous spinal stimulation can improve hand and arm function after SCI [10]. More recently, a multicenter trial showed that non-invasive cervical spinal stimulation paired with rehabilitation can produce clinically meaningful gains in upper-extremity strength and function in chronic tetraplegia [11]. For the upper limb, sensing may be as important as stimulation. Ting et al. showed that wearable sleeve arrays can decode neural drive from paralyzed muscles during attempted movement [31,36]. Oliveira et al. extended this concept by using a direct spinal cord–computer interface to enable control of a paralyzed hand, illustrating how high-information biosignals can be converted into functional motor commands even in severe SCI [32]. Together, these studies suggest a practical architecture for future cervical neuromodulation: detect residual intent with EMG or related biosignals, then use that intent to gate or shape stimulation during grasp, release, or reaching. Compared with lower-limb applications, upper-limb systems will likely require denser sensing, greater anatomical selectivity, and more individualized calibration, but the same translational rule still applies: clinically useful systems are unlikely to depend on a single perfect signal.

## 7. Translational Challenges and Future Directions

Across gait, balance, and upper-limb applications, the same technical barriers recur. First, signals are unstable. EMG varies with electrode placement, perspiration, spasms, and fatigue. Kinematic and pressure signals drift with assistive devices, footwear, and training context. Even invasive systems must contend with day-to-day biological variability [20,21,31]. Second, real-time control remains demanding. A model that performs well offline may still fail if latency is too high or if confidence drops during transitions between tasks. This is why many successful SCI systems still rely on simple, interpretable control rules such as phase-triggered stimulation or threshold-based intent detection. Edge-AI approaches are promising precisely because they aim to preserve low latency while extending functionality [16,20]. Third, SCI is heterogeneous. Injury level, chronicity, spared sensory pathways, autonomic involvement, and coexisting orthopedic limitations all influence controller performance. A strategy that works in treadmill stepping with body-weight support may not transfer directly to community walking or home use [37]. Recent reviews therefore emphasize multimodal sensing, personalized control policies, and closer integration of neuromodulation with robotics and rehabilitation training [12].

Not all stimulation paradigms need the same level of feedback control. In some rehabilitation settings, stimulation mainly serves to increase spinal excitability while the task itself is constrained by the therapist, treadmill, body-weight support system, or training protocol. Under those conditions, continuous stimulation, pre-programmed stimulation, or therapist-triggered stimulation may be sufficient, particularly during early mapping, seated training, or repetitive treadmill stepping. Closed-loop control becomes more important when the timing of stimulation determines whether the response is useful. For example, stance-to-swing transitions, start and stop commands, changes in cadence, perturbations during standing, and the onset or release phase of grasp. In these settings, stimulation that arrives too early or too late may not simply be ineffective; it may interfere with the intended movement or reduce stability. Thus, the goal should not be to build the most complex controller possible, but to match the degree of feedback control to the task, the patient, and the clinical risk.

This distinction is important because the sensor errors propagate directly into stimulation timing. EMG signals can change with electrode placement, sweating, stimulation artifact, fatigue, spasticity, and skin impedance. Kinematic sensors may drift or shift when attached to braces, clothing, walkers, or orthotic devices. Pressure and force sensors are sensitive to footwear, partial loading, and uneven weight distribution. Vision-based systems can provide rich movement information, but their performance depends on lighting, camera position, occlusion, privacy constraints, and available processing power. These problems are not always apparent in offline analyses. A classifier may perform well on stored data but still be clinically unreliable if the delay from sensing to stimulation is too long, if transitions are missed, or if the system behaves unpredictably when a sensor signal drops out. Future studies should therefore report practical control metrics, including end-to-end latency, calibration time, false triggers, missed events, session-to-session stability, and the behavior of the system during sensor failure.

SCI heterogeneity is not just a clinical descriptor. It changes which control signal is available. Individuals with SCI differ in injury level, chronicity, residual descending drive, preserved proprioception, spasticity, autonomic dysfunction, pain, and orthopedic limitations. A controller based on residual EMG may work well in a patient with detectable attempted movement but be unsuitable for someone without reliable peripheral muscle signals. A pressure- or kinematic-based controller may be adequate for rhythmic stepping, yet provide little information about hand intent or fine upper-limb control. For this reason, future systems will likely need hybrid designs rather than a single universal control strategy. A practical architecture may combine tonic or task-specific stimulation to establish a permissive excitability state, event-triggered modulation for movements that require precise timing, and conservative fallback modes when sensor confidence is low. In this framework, closed-loop control is valuable not because it is technologically sophisticated, but because it reduces the specific uncertainties that limit safe and effective function in a given patient.

SCI heterogeneity is not just a clinical descriptor. It changes which control signal is available. Individuals with SCI differ in injury level, chronicity, residual descending drive, preserved proprioception, spasticity, autonomic dysfunction, pain, and orthopedic limitations. A controller based on residual EMG may work well in a patient with detectable attempted movement, but is unsuitable for someone without reliable peripheral muscle signals. A pressure- or kinematic-based controller may be adequate for rhythmic stepping. Yet provide little information about hand intent or fine upper-limb control. For this reason, future systems will likely need hybrid designs rather than a single universal control strategy. In practical terms, this may involve using one or more safety measures, such as foot pressure, limb kinematics, or trunk orientation; disagreement among these signals could trigger a conservative fallback mode. A practical architecture may combine tonic or task-specific stimulation timing and conservative fallback modes when sensor confidence is low. In this framework, closed-loop control is valuable not because it is technologically sophisticated, but because it reduces the specific uncertainties that limit safe and effective function in a given patient.

For sensor-focused translation, future studies should report placement, latency, calibration time, dropout handling, and cross-session stability. Animal studies will continue to clarify the mechanism and test new control logic, whereas clinical work should increasingly emphasize portability, standardization of reporting, and clinically meaningful endpoints [6,7,11,14,15,16,20]. The most successful next-generation systems may not be the most complex ones; they may be the ones that combine simple sensing, reliable control, and enough adaptability to be used every day.

## 8. Conclusions

Closed-loop neuromodulation has moved SCI rehabilitation from fixed stimulation toward adaptive, sensor-guided motor restoration. Animal studies established the value of activity-dependent timing, proprioceptive engagement, and anatomically selective recruitment, while clinical studies support the feasibility that these principles can potentially improve stepping, walking, standing, trunk control, and upper-limb function [3,5,8,11,20].

The major remaining challenge is no longer whether stimulation can activate the injured nervous system, but how to sense motor state robustly enough to drive stimulation in real time. The main implication is that stimulation hardware alone is insufficient; sensing and control design will determine whether these systems work outside the laboratory.

## 9. Limitations of This Review

This review remains narrative and was not designed as a systematic review or meta-analysis. It does not claim exhaustive capture of every relevant paper, and no formal risk-of-bias tool was applied. The evidence classification is descriptive and is intended to clarify study type and translational maturity rather than to grade intervention efficacy.

## Figures and Tables

**Table 1:** Representative animal and clinical studies highlighted in this review.

Domain/Study	n/Sample/Model; Evidence Level	Algorithm/Controller; Timing/Calibration	Control Type; Sensor/Estimated Variable	Stimulation/Interface	Functional Endpoint/Main Finding	Refs.
Gait	Chronic cervical SCI rat	Triggered spinal stimulation paired with detected activity; threshold/delay calibration described; standardized latency metrics limited/NR	Activity-dependent movement-related signals	Targeted spinal stimulation; invasive	Long-lasting motor recovery with activity-dependent timing	[6]
Gait	Severe SCI rat	Predefined spatiotemporal stimulation pattern engaging muscle synergies; online sensor-control latency NR	Task-specific open-loop/contextual; spatiotemporal gait-state program	Lumbar neuromodulation; invasive	Refined locomotor control through stimulation patterns engaging muscle synergies; preclinical translational/contextual evidence	[4]
Gait	SCI primate	Brain–spine interface triggering spinal stimulation; decoder calibration required; latency not uniformly reported	Event-triggered; motor-contex activity/leg-movement intent	Brain–spine interface; intracortical recording + epidural stimulation; invasive	Real-time cortical triggering alleviated gait deficits; preclinical translational evidence	[13]
Gait/balance	Severe SCI rat	Robotic feedback control of trunk posture; timing controlled by posture state; not electrical neuromodulation	Closed-loop posture control/contextual; trunk posture feedback	Closed-loop robotic postural control; non-simulation mechanistic context	Improved stepping by rebalancing proprioceptive feedback; mechanistic contextual evidence	[14]
Gait	SCI rat	Proportional brain–spine interface modulating stimulation amplitude; minimal calibration reported; latency NR	Real closed-loop; cortical ensemble activity/swing-related motor state	Proportional brain–spine interface; intracortical + epidural stimulation; invasive	Improved immediate locomotion and long-term recovery; preclinical translational evidence	[15]
Gait	SCI mouse	Edge-AI classifier generating phase-dependent stimulation triggers; on-device real-time inference reported; formal latency validation limited	Event-triggered; video-derived joint angles/gait phase	Phase-dependent closed-loop neuromodulation; camera + external controller + stimulation hardware; hybrid inference reported; formal latency validation limited	Real-time gait-phase triggering on embedded hardware; preclinical proof-of-concept evidence	[16]
Gait	Complete paraplegia; one adult	Task-linked clinical programming selected before or during therapy; no closed-loop latency	Continuous stimulation + training/contextual; no online control sensor	Lumbosacral epidural stimulation; invasive	Independent stepping after complete paraplegia; human case report/contextual evidence	[17]
Gait	Chronic motor-complete SCI; four adults	Intensive training with tailored epidural programming; no online sensor-controller loop	Continuous stimulation + training/contextual; no online control sensor	Epidural stimulation; invasive	Overground walking recovered in selected individuals; human case series/contextual evidence	[18]
Gait	Chronic SCI; three adults	Individualized spatiotemporal programs linked to gait events; individual calibration required; standardized latency reporting limited	Event-triggered; gait state/stepping phase	Targeted epidural stimulation; invasive	Adaptive overground walking restored within days; human case series	[5]
Gait/multi-task	Complete paralysis; three adults	Activity-specific task programs selected for each behavior; online sensor control absent	Task-specific open-loop/contextual; no online control sensor	Epidural stimulation; invasive	Rapid restoration of standing, walking, cycling, swimming, and trunk control; human case series/contextual evidence	[7]
Gait	Chronic tetraplegia; one adult	Decoder-driven brain–spine interface selecting stimulation programs; regular recalibration described; near-real-time use	Real closed-loop; cortical implants/intended leg movement	Brain–spine interface; implanted cortical recording + epidural stimulation; invasive	Natural walking in community settings; first in-human case report	[8]
Gait	Paraplegia; two adults	BMI-triggered multichannel surface FES; training and decoder calibration required; latency NR	Real closed-loop; non-invasive brain intent/motor imagery	Brain-controlled FES; non-invasive/hybrid	Feasibility of non-invasive intent-driven locomotor rehabilitation; human feasibility evidence	[19]
Gait	Paraplegia; human participants	Thresholded hand-EMG-triggered magnetic spinal stimulation; threshold calibration described; latency NR	Real closed-loop; hand muscle EMG/volitional stepping command	Non-invasive lumbar magnetic stimulation; non-invasive	Control of start-stop stepping, cadence, and step length; early human feasibility evidence	[20]
Walking rehab	Motor-incomplete SCI; clinical cohort	tSCS delivered with walking therapy; no online sensor-triggered control	Clinical rehabilitation without control sensor/contextual; clinical gait metrics only	tSCS + walking therapy; non-invasive	Feasible adjunct to walking-based rehabilitation; prospective contextual evidence	[21]
Walking rehab	Subacute SCI; randomized feasibility cohort	tSCS or sham paired with locomotor training; no closed-loop latency	Clinical rehabilitation without control sensor/contextual; clinical walking outcomes only	tSCS + locomotor training; non-invasive	Feasibility and efficacy for walking and spasticity; randomized feasibility/contextual evidence	[22]
Walking rehab	Motor-incomplete tetraplegia; single-subject design	Multisite stimulation strategy paired with training; programming reported; online sensor control absent	Task-specific/continuous stimulation without an online control sensor	tSCS; non-invasive	Walking and autonomic improvements; single-subject/contextual evidence	[23]
Seated/standing balance	SCI; implanted neuroprosthesis users	Threshold or feedback controller driving hip/trunk extensor stimulation; calibration described; latency NR	Real closed-loop; trunk tilt/posture thresholds	FNS neuroprosthesis; external posture sensor + implanted stimulation; hybrid invasive	Improved seated posture and balance control; human feasibility evidence	[24,25,26,27]
Trunk/standing	SCI; clinical cohorts	Non-invasive spinal stimulation during trunk or standing tasks; closed-loop metrics NR	Continuous or task-specific stimulation without online control of the sensor/contextual	Non-invasive spinal stimulation; non-invasive	Improved trunk stability and self-assisted standing; pilot/contextual evidence	[28,29]
Upper limb	SCI primate	Phase-specific cervical dorsal root stimulation; phase calibration reported; latency NR	Event-triggered/phase-specific; movement phase + segmental recruitment	Cervical epidural stimulation; invasive	Restored voluntary reach-and-grasp control; preclinical translational evidence	[9,30]
Upper limb	Tetraplegia; clinical studies	Transcutaneous cervical stimulation paired with hand/arm rehabilitation; session programming reported; no real-time controller	Task-specific open-loop/rehabilitation without a real-time control sensor	Transcutaneous cervical stimulation; non-invasive	Improved hand and arm function in chronic cervical SCI; clinical contextual evidence	[10,11]
Upper limb	Tetraplegia; sensor/control-signal studies	Intent decoding for hand control; calibration required; stimulation-control latency depends on platform/NR	Contextual sensor evidence; sleeve array or direct spinal cord-computer interface	Sensor/interface platforms; non-invasive sleeve array or spinal interface, depending on study	Residual neural drive can be sensed and converted into functional hand control; sensor proof-of-concept/contextual evidence	[31,32]

Note: NR indicates not reported. “Contextual” evidence indicates studies included for translational relevance but not counted as direct proof of real-time closed-loop sensor-stimulation control.

**Table 2:** Major sensor classes used in closed-loop neuromodulation for spinal cord injury.

Sensor Class	Typical Variable	Common Control Use	Strengths	Limitations	Representative Refs.
Kinematic sensors (IMUs, goniometers, encoders, motion capture)	Joint angle, limb trajectory, trunk orientation	Gait phase detection; step timing; posture tracking	Portable; intuitive; low computational burden	Drift; calibration burden; placement sensitivity	[5,7,21,23]
Force/pressure sensors (foot switches, insoles, force plates, load cells)	Foot contact, stance loading, weight shift, center of pressure	Stance-swing detection; standing balance; weight-shift control	Reliable event detection; simple thresholds	Footwear/device dependence; limited intent information	[24,25,26,28,29]
Surface EMG	Muscle activation amplitude and timing	Intent gating; adaptive stimulation; response monitoring	Physiologically meaningful; widely available	Noise; cross-talk; spasticity and motion artifact	[10,20]
EMG/sleeve arrays	Residual motor-unit activity and neural drive	Fine upper-limb intent decoding; virtual or neuroprosthetic control	High information content; useful in severe weakness	More setup and processing; session-to-session variability	[31,32]
Vision-based sensing	Video-derived joint kinematics and posture	Markerless gait phase; laboratory and potential home monitoring	Low-contact; rich spatial information	Occlusion; lighting sensitivity; privacy concerns; processing load	[16]
Cortical or neural interfaces	Cortical ensemble activity, brain intent signals	Brain–spine or brain–FES control	Direct access to volitional intent	Invasive or low-SNR, depending on modality	[8,9,15,19]

Abbreviations: EMG, electromyography; FES, functional electrical stimulation; IMU, inertial measurement unit; SCI, spinal cord injury; tSCS, transcutaneous spinal cord stimulation.

## Data Availability

No new data were created or analyzed in this review.
